# Supplementary material for: Atlantic deep water provenance decoupled from atmospheric CO2 concentration during the lukewarm interglacials
Source: Nat Commun. 2017 Dec 8;8:2003. doi: 10.1038/s41467-017-01939-w (PMC5722826; doi:10.1038/s41467-017-01939-w)
Supplement: Supplementary file 2 — Description of Additional Supplementary Files [file 41467_2017_1939_MOESM2_ESM.pdf]

**Description of Additional Supplementary Files**

File Name: Supplementary Data 1
Description: Radiocarbon data used in constructing the age model of the upper section of ODP 929

File Name: Supplementary Data 2
Description: Additional benthic foraminiferal stable isotope data measured for ODP 929

File Name: Supplementary Data 3
Description: Authigenic neodymium isotope measurements for ODP 929
